# Awareness of diagnosis, treatment and risk of late effects in Chinese survivors of childhood cancer in Hong Kong

**DOI:** 10.1111/hex.13288

**Published:** 2021-06-08

**Authors:** Lok Sum Yang, Chung Tin Ma, Chun Him Chan, Mei Shum Luk, Hoi Kei Woo, Vivian Wai‐yan Lee, Alex Wing Kwan Leung, Samantha Lai‐ka Lee, Nelson Chun‐yiu Yeung, Chi‐kong Li, Yin Ting Cheung

**Affiliations:** ^1^ School of Pharmacy Faculty of Medicine The Chinese University of Hong Kong Hong Kong Hong Kong; ^2^ Centre for Learning Enhancement and Research The Chinese University of Hong Kong Hong Kong Hong Kong; ^3^ Department of Paediatrics & Adolescent Medicine Hong Kong Children’s Hospital Hong Kong Hong Kong; ^4^ Department of Paediatrics Faculty of Medicine The Chinese University of Hong Kong Hong Kong Hong Kong; ^5^ The Jockey Club School of Public Health and Primary Care Faculty of Medicine The Chinese University of Hong Kong Hong Kong Hong Kong; ^6^ Hong Kong Hub of Paediatric Excellence The Chinese University of Hong Kong Hong Kong Hong Kong

**Keywords:** childhood cancer, education, health literacy, late effects, survivorship

## Abstract

**Background:**

For survivors of childhood cancer, awareness of personal health risks is a critical component of long‐term health management.

**Objective:**

To evaluate the awareness of the diagnosis, treatment and risk of late effects among survivors of childhood cancer in Hong Kong.

**Methods:**

Between June 2019 and March 2020, this cross‐sectional study recruited 155 adult survivors (mean age = 26.9, standard deviation [SD] = 6.4 years) and 45 parents of paediatric survivors (mean age = 11.1, SD = 3.6 years) from a long‐term follow‐up clinic. At >10 years post‐treatment (mean = 13.4, SD = 7.6 years), they completed a structured questionnaire to report their cancer‐specific knowledge. Multiple linear regression analysis was conducted to identify clinical, socioeconomic and behavioural factors associated with poor awareness.

**Results:**

The majority of participants accurately recalled their diagnoses (73.5%) and major treatment modalities (chemotherapy 92.4%, radiation 82.9% and surgery 88.2%). However, less than half (45%) of the participants recognized more than 25% of the total late effects for which they were at risk. The highest levels of awareness were reported for endocrine problems (49%), neurocognitive impairment (44%) and secondary cancers (43%), and the lowest for peripheral neuropathy (21%) and vision problems (23%). Compared with survivors of haematological malignancies, those of central nervous system (CNS) tumours (standardized estimate [B] = −9.33, 95% confidence interval [95% CI]: −13.41 to −5.26) and non‐CNS solid tumours (*B* = −8.47, 95% CI: −12.39 to −4.94) had less knowledge about their diagnosis. Retaining medical records (*P* < .0001) and better medical information‐seeking habits (*P* = .048) were associated with better awareness.

**Conclusions:**

Survivors of childhood cancer in Hong Kong have deficient awareness of their personal health risks. They may benefit from the provision of a survivorship care plan and personalized education regarding treatment‐related late effects.

**Patient Contribution:**

Patients contributed in designing the study tools. Results were presented at a non‐governmental organization.

## 1 INTRODUCTION

Between 2014 and 2018, there is an average of 180 paediatric patients (<19 years old) newly diagnosed with cancer each year in Hong Kong.^1^ Similar to most developed countries, contemporary treatment strategies have led to a dramatic reduction in the late mortality of childhood cancers, hence yielding an emerging population of childhood cancer survivors.^2^ However, treatment modalities for childhood cancers, including chemotherapy, radiotherapy, surgery and haematopoietic stem cell transplantation (HSCT), are associated with late effects that can reduce the quality of life of survivors. The high prevalence of these late effects was illustrated by the results of one landmark study in the United States, in which authors observed an 80.5% estimated cumulative prevalence of serious/disabling or life‐threatening chronic conditions in childhood cancer survivors by the age of 45 years.^3^ Survivors also tended to have more problems with physical functioning associated with treatment‐related late effects when compared with their healthy counterparts.^4^, ^5^ These observations have led researchers and medical professionals to focus on enhancing the quality of life of childhood cancer survivors in recent years.

One goal of quality survivorship care is to improve the health literacy of survivors by helping them understand the nature, screening, prevention and management of possible late effects.^6^ The World Health Organization defines health literacy as ‘the cognitive and social skills which determine the motivation and ability of individuals to gain access to, understand and use information in ways which promote and maintain good health’.^7^ Studies comparing general and cancer populations have revealed associations of a low level of health literacy with increased medical costs, poor medication adherence, the misinterpretation of health information, a lower level of physical activity and a higher risk of developing health‐damaging habits, such as smoking.^8^, ^9^ Conversely, increased awareness of the symptoms of potential late effects in cancer survivors can prompt preventive action and timely management.^10^ For instance, a Chinese study observed that more health‐literate survivors of cancer tend to have better survivorship outcomes, including cognitive and social functioning, mental well‐being and financial ability.^11^ Empowering the health literacy and cancer‐specific knowledge of survivors is therefore beneficial in terms of improving their quality of life.

Several studies in Western countries have revealed that a younger age at diagnosis, a lower socioeconomic status (SES) and the specific cancer type are the major factors influencing health literacy among survivors and caregivers.^12^, ^13^ However, these risk factors may not be generalizable to the Asian population due to differences in cultural, lifestyle and health practices.^14^ To date, no study has evaluated the cancer‐specific knowledge held by survivors of childhood cancer in Hong Kong. Identifying the knowledge gaps in this population might facilitate the development of interventions to empower survivors in managing their long‐term health.

The primary objective of this study was to examine the extent of knowledge regarding the diagnosis, treatment and risk of late effects in survivors of childhood cancer in Hong Kong and to identify the predictors of cancer‐specific health literacy in this population.

## 2 METHODS

### 2.1 Study design and setting

This was a cross‐sectional observational study. The study protocol was approved by the Joint Chinese University of Hong Kong—New Territories East Cluster Clinical Research Ethics Committee (reference number 2018.338). Survivors of childhood cancer were recruited via consecutive sampling from June 2019 to March 2020 at the Long‐term Follow‐up (LTFU) Clinic of the Prince of Wales Hospital in Hong Kong. This regional tertiary care public hospital serves as a major hub to provide LTFU care to survivors of childhood cancer. Written informed consent was obtained from all participants.

### 2.2 Subjects

Survivors were eligible for inclusion if they were diagnosed with cancer by a paediatric oncologist/haematologist before 18 years of age, received cancer treatment at the study institution and had been in remission for ≥5 years post‐diagnosis. Patients who had a cancer relapse or secondary malignancy were also included in this study if they were ≥2 years post‐treatment (ie chemotherapy, surgery or radiation treatment) at the time of the interview. The survivor's parent participated in the survey if the survivor was younger than 16 years or cognitively impaired. Participants who were not able to understand the Chinese language were excluded.

### 2.3 Data collection method

Adult survivors and parents of paediatric survivors (referred as ‘participants’ hereafter) completed a structured questionnaire to evaluate their cancer‐specific knowledge and medical information‐seeking habits (Appendix S1).

### 2.4 Primary outcome: cancer‐specific health literacy

The approach used to assess cancer‐specific health literacy was adapted from three landmark studies (ie Landier et al, Kadan‐Lottick et al and Syed et al).^13^, ^15^, ^16^ The assessment referred to survivors’ knowledge in three specific fields. (1) *Cancer diagnosis*. Participants were asked to report their cancer diagnoses in as much detail as possible, including the type, site and stage of their primary cancer. (2) *Treatment history*. Participants were asked to report the major treatment modalities they received (chemotherapy, radiotherapy, surgery and/or HSCT), radiation sites and surgery sites. (3) *Risk‐based, exposure‐related late effects*. Personal health risks were determined based on the Children's Oncology Group (COG)‐LTFU Guidelines.^17^ The COG‐LTFU guideline is a personalized systematic programme of regular screening, surveillance and prevention strategies based on each survivor's cancer type, treatment regimen and cancer experience.^6^, ^17^ Each survivor's responses regarding their awareness of health risks were compared with their actual exposure‐related health risks for 12 risk‐based, exposure‐related complications that are highly prevalent and/or associated with potentially serious health consequences and/or known to adversely affect functional outcomes, according to the current literature (Table 1).^3^, ^5^, ^6^, ^14^, ^18^ These complications include organ‐specific problems (pulmonary, cardiac, hepatic, musculoskeletal, vision, hearing), secondary malignancy, neurocognitive impairment, infertility, peripheral neuropathy and endocrine problems. The participant's overall awareness of health risks was defined as the proportion of at‐risk complications that were correctly identified by him/her. For example, a participant who was previously treated with methotrexate is at risk for musculoskeletal problems, hepatic problems and neurocognitive impairment (a total of 3 at‐risk complications); if he were to be able to identify 2 of the 3 health risks, his overall awareness would be graded as 66.6%.

**TABLE 1:** Treatment‐related late effects

Late effect	Therapeutic exposures^a^	Expected development timeline
Secondary malignancy	RT: any Chemotherapy: alkylating agents, epipodophyllotoxins, anthracycline Surgery: HSCT Any cancers	Late
Renal problems	RT: upper abdomen, flank, para‐aortic, TBI Chemotherapy: ifosfamide, cisplatin, carboplatin, methotrexate Surgery: HSCT, nephrectomy, cystectomy Wilm's tumour, cancers of the kidney and bladder	Early
Musculoskeletal problems	RT: spine, hips, lower limbs Chemotherapy: corticosteroids, methotrexate, alkylating agents (high dose) HSCT Sarcoma cancers	Any time point
Peripheral neuropathy	Chemotherapy: vincristine, vinblastine, cisplatin, carboplatin Cancers of the central nervous system or peripheral nervous system	Early
Cardiac problems	RT: chest, mantle, mediastinal, lung, spine, left kidney, whole abdomen, TBI Chemotherapy: doxorubicin, daunorubicin, idarubicin, epirubicin, mitoxantrone HSCT	Late
Infertility	RT: pelvis, lower spine, testicular, neuroendocrine axis, TBI Chemotherapy: all alkylating agents, cisplatin, carboplatin, oxaliplatin Surgery: oophorectomy, orchiectomy Cancers of the central nervous system (CNS tumours), germ cell tumours	Early
Hepatic problems	RT: liver, whole abdomen, TBI Chemotherapy: methotrexate, mercaptopurine, dactinomycin, thioguanine Surgery: hepatectomy HSCT Cancers of the liver	Late
Vision problems	RT: head/face, orbital, cranium, TBI Chemotherapy: busulfan, corticosteroids Surgery: eye surgery Cancers of the eye (retinoblastoma) or the head or the central nervous system	Early
Hearing problems	RT: cranium, ear/infratemporal, nasopharyngeal region, Waldeyer's ring Chemotherapy: cisplatin, carboplatin Cancer of the head or the central nervous system	Early
Neurocognitive impairment	RT: brain, craniospinal, head Chemotherapy: high‐dose cytarabine, high‐dose methotrexate Surgery: neurosurgery Cancer of the head or the central nervous system	Any time point
Endocrine problems	RT: cranium, eye, ear, naso‐oropharyngeal, chest, cervical/neck‐spine, mantle, TBI, systemic metaiodobenzylguanidine therapy, pelvis, lower spine, testicular, neuroendocrine axis Surgery: brain, removal of pituitary gland, oophorectomy, orchiectomy Cancer of the central nervous system, germ cell tumours	Early
Pulmonary problems	RT: lungs, chest, chest, mantle, mediastinal, lung, spine, whole abdomen, TBI Chemotherapy: busulfan, carmustine, lomustine, bleomycin	Early

Note

HSCT, haematopoietic stem cell transplant; RT, radiation therapy; TBI, total body irradiation.

^a^ Exposure‐related health risks were determined according to the Children's Oncology Group Long‐term Follow‐up Guidelines. ‘Early onset’ refers to late effects that may occur within 5 to 10 y post‐diagnosis, while ‘late onset’ refers to late effects that may occur at >10 y post‐diagnosis.^17^

To assess the accuracy of the participants’ reports, their responses were verified against information abstracted from the Clinical Management System (CMS), an electronic health data repository of the public health‐care system in Hong Kong that is considered a reliable data source for epidemiological research in Hong Kong.^19^ The CMS includes cancer‐related variables (eg diagnoses and age at diagnosis) and treatment‐related variables (eg treatment start and end dates, treatment protocols, chemotherapy drugs, surgical sites and radiation sites).

The responses were then graded according to the scoring system presented in Appendix S2. Three subscores (diagnosis awareness score, treatment awareness score and late effects awareness score) were computed (range: 0 to 100), with higher scores indicative of better knowledge.

### 2.5 Predictors and covariates

Factors associated with the above outcomes were categorized into clinical, SES and behavioural factors. Clinical factors included the age at diagnosis, time elapsed since treatment completion, primary cancer diagnosis (haematological malignancy vs central nervous system [CNS] tumour vs non‐CNS solid tumour), relapse status and diagnosis of a chronic health condition. Information on chronic health conditions was collected through self‐ or proxy reports and further verified with the Statistical Classification of Diseases and Related Health Problems [ICD]‐9 codes and doctors’ consultation notes registered on the CMS, or medications in dispensing records. Only health conditions with an age of onset after the completion of treatment were included in the analysis.

Socioeconomic status factors included the self‐reported household monthly income (less than vs more than HKD 30 000 [USD 3850]), housing type (public vs private, which are typical markers of low and high SES, respectively, in Hong Kong) and private health insurance possession (yes vs no). For adult survivors, the current employment status (employed full‐time vs not full‐time) and the highest educational attainment (≤ secondary school vs > secondary school) were also used as surrogate markers of SES.

Behavioural factors were those related to habitually keeping medical records and medical information‐seeking ability. Participants were asked whether they had systematically kept documentation of the survivor's medical records, such as discharge summaries, treatment records and laboratory reports provided by clinicians. Medical information‐seeking ability was assessed using the traditional Chinese version of the Health Literacy Questionnaire (HLQ),^20^ which has demonstrated good internal consistency and reliability in evaluations of patients with various medical conditions^21^ and within the Chinese population.^22^ For this study, five questions were extracted from the ‘appraisal of health information’ subscale to assess each participant's ability to compare health information from different sources, decide the best health information for their needs, evaluate the accuracy and reliability of new health information and seek advice from health‐care professionals regarding the quality of the health information. Each item was rated on a 4‐point Likert scale from 1 (strongly disagree) to 4 (strongly agree), and the item scores were summed to yield a total score ranging from 1 to 20 points. A higher score indicates a better ability to acquire and interpret health‐related information.

### 2.6 Statistical analysis

Descriptive statistics are used to summarize the demographic and clinical characteristics of the participants. First, a univariate analysis was conducted to identify the factors associated with the following primary outcomes: (1) diagnosis awareness score, (2) treatment awareness score and (3) late effects awareness score. The Mann–Whitney U test was used to evaluate independent variables that are categorical in nature (eg education level, cancer diagnosis, presence of chronic health condition). The Spearman correlation test was applied to continuous variables (eg age at diagnosis, time since treatment and HLQ score). Factors that were significantly associated (*P* < .10) with any of the three subscales in the univariate analyses were included in subsequent multivariable analyses.

Multiple regression models were used to identify factors associated with the primary outcomes after adjustment for the covariates of sex and age at evaluation. These associations are presented using standardized estimates (B) and 95% confidence intervals (CIs). The significance threshold was set at *P* < .05. As the responses for paediatric patients (<16 years of age) were proxy‐reported by their parents, a sensitivity analysis was conducted by repeating the multivariable analysis in adult survivors only (ie on self‐reported data only).

All statistical analyses were performed using SAS (SAS 9.4, SAS Institute, Cary, NC, USA) and were two‐tailed.

## 3 RESULTS

### 3.1 Participant demographics

Two hundred and fifty‐two survivors were screened for eligibility, of whom 39 were excluded because less than 2 years had elapsed post‐treatment or they had received a non‐cancer diagnosis (eg benign ovarian teratoma) (Appendix S3). Subsequently, 213 eligible participants were approached; 205 eligible survivors agreed to participate in the study, and 8 declined to participate. Five participants were further excluded because of incomplete treatment records. Finally, the data of 200 participants (155 adult survivors and 45 paediatric survivors) were analysed (response rate: 93.9%) (Table 2).

**TABLE 2:** Characteristics of study population (n = 200)

Sample characteristics	All survivors N = 200	Adult survivors n = 155	Paediatric survivors n = 45	Parents^a^ N (%) n = 45
**Demographic and clinical characteristics**	**Mean [SD]**	**Mean [SD]**	**Mean [SD]**	**Mean [SD]**
Age at Interview [y]	23.4 [8.8]	26.9 [6.4]	11.1 [3.6]	43.4 [7.7]
Age of Diagnosis [y]	7.3 [5.2]	8.61 [4.9]	2.8 [2.5]	
Years From Treatment Completion [y]	13.4 [7.6]	15.4 [7.3]	6.8 [3.4]	
	**n (%)**	**n (%)**	**n (%)**	**n (%)**
Sex				
Male	110 (55.0)	86 (55.5)	24 (53.3)	31 (68.9)
Female	90 (45.0)	69 (44.5)	21 (46.7)	14 (31.1)
Diagnosis^b^				
Haematological malignancy				
Leukaemia	78 (39.0)	64 (41.3)	14 (31.1)	
Lymphomas	28 (14.0)	24 (15.5)	4 (8.9)	
CNS tumour	14 (7.0)	11 (7.1)	3 (6.7)	NA
Non‐CNS solid tumour				
Neuroblastoma	13 (6.5)	6 (3.9)	7 (15.6)	
Retinoblastoma	2 (1.0)	2 (1.3)	0 (0)	
Renal tumour	10 (5.0)	8 (5.2)	2 (4.4)	
Hepatic tumour	7 (3.5)	2 (1.3)	5 (11.1)	
Bone tumour	18 (9.0)	16 (10.3)	2 (4.4)	
Soft tissue sarcomas	14 (7.0)	9 (5.8)	5 (11.1)	
Germ cell tumour	11 (5.5)	8 (5.2)	3 (6.7)	
Others^c^	5 (2.5)	5 (3.2)	0 (0)	
History of Cancer Relapse	31 (15.5)	26 (16.8)	5 (11.1)	
Presence of Chronic Health Condition				
No	117 (58.5)	90 (58.1)	27 (60.0)	
Yes				
1 condition	46 (23.0)	37 (23.9)	9 (20.0)	
2‐4 conditions	32 (16.0)	23 (14.8)	9 (20.0)	
5‐6 conditions	5 (2.5)	5 (3.2)	0 (0)	
**Socioeconomic characteristics**	n (%)	n (%)	n (%)	n (%)
Education Level				
Secondary school or below	92 (46.0)	48 (31.0)	44 (97.8)	29 (64.4)
Post‐secondary school or above	107 (53.5)	107 (69.0)	0 (0)	16 (35.6)
Special education	1 (0.5)	0 (0)	1 (2.2)	0 (0)
Employment Status				
Student	88 (44.0)	43 (27.7)	45 (100)	0 (0)
Full‐time Employment	86 (43.0)	86 (55.5)	0 (0)	25 (55.6)
Part‐time Employment	13 (6.5)	13 (8.4)	0 (0)	8 (17.8)
Unemployed/ job transition	8 (4.0)	8 (5.2)	0 (0)	0 (0)
Housewife	5 (2.5)	5 (3.2)	0 (0)	1 (2.2)
Retired	0 (0)	0 (0)	0 (0)	11 (24.4)
Private Medical Insurance				
Yes	86 (43.0)	63 (40.6)	23 (51.1)	
No	113 (56.5)	91 (58.7)	22 (48.9)	
*Missing*	1 (0.5)	1 (0.6)	0 (0)	
Monthly Household Income (in HKD)				
<$15,000	32 (16.0)	20 (12.9)	12 (26.7)	
$15,001‐$30,000	58 (29.0)	46 (29.7)	12 (26.7)	
$30,001‐$50,000	42 (21.0)	33 (21.3)	9 (20.0)	
>$50,000	60 (30.0)	49 (31.6)	11 (24.4)	
*Missing*	8 (4.0)	7 (4.5)	1 (2.2)	
Housing				
Public Housing (includes Public Rental Housing and Subsidized Home Ownership Housing)	103 (51.5)	78 (50.3)	25 (55.6)	
Private Housing	78 (39.0)	63 (40.7)	15 (33.3)	
Others	18 (9.0)	13 (8.4)	5 (11.1)	
*Missing*	1 (0.5)	1 (0.6)	0 (0)	
**Treatment characteristics**	n (%)	n (%)	n (%)	n (%)
Chemotherapy				
None	15 (7.5)	10 (6.5)	5 (11.1)	
Any	185 (92.5)	145 (93.5)	40 (88.9)	
Alkylating agents	122 (61.0)	95 (61.3)	27 (60.0)	
Anthracyclines	136 (68.0)	108 (69.7)	28 (62.2)	
Antimetabolites	115 (57.5)	92 (59.4)	23 (51.1)	
Enzymes	62 (31.0)	51 (32.9)	11 (24.4)	
Podophyllotoxins	98 (49.0)	78 (50.3)	20 (44.4)	
Plant Alkaloids	125 (62.5)	100 (64.5)	25 (55.6)	
Platinum Agents	54 (27.0)	38 (24.5)	16 (35.6)	
Corticosteroids	109 (54.5)	87 (56.1)	22 (48.9)	
Others	57 (28.5)	46 (29.7)	11 (24.4)	
Radiation				
None	130 (65.0)	94 (60.6)	36 (80.0)	
Any	70 (35.0)	61 (39.4)	9 (20.0)	
Cranial	36 (18.0)	34 (21.9)	2 (4.4)	
Others	34 (17.0)	27 (17.4)	7 (15.6)	
Surgery				
None	115 (57.5)	93 (60.0)	22 (48.9)	
Any	85 (42.5)	62 (40.0)	23 (51.1)	
Neurosurgery	14 (7.0)	10 (6.5)	4 (8.9)	
Others	71 (35.5)	52 (33.5)	19 (42.2)	
HSCT	30 (15.0)	22 (14.2)	8 (17.8)	
**Behavioural characteristics**	n (%)	n (%)		n (%)
Personal medical record‐keeping habit^d^	106 (53.0)	83 (53.5)		23 (51.1)
	**Mean [SD]**	**Mean [SD]**		**Mean [SD]**
Medical information‐seeking ability^e^	13.8 [2.7]	13.8 (2.9)		13.9 (1.7)

Abbreviations: CNS, central nervous system; HSCT, haematopoietic stem cell transplant; NA, not applicable.

^a^ Parents completed the questionnaire on behalf of paediatric survivors (age ≤16 y).

^b^ Classified according to International Classification of Childhood Cancer.

^c^ Other cancer types included adrenal gland carcinoma, nasopharyngeal carcinoma, primary adnexal carcinoma, mucoepidermoid carcinoma and adrenocortical carcinoma.

^d^ Refers to systematic documentation of discharged summaries, treatment records and laboratory reports provided by clinicians.

^e^ Refers to the ‘Appraisal of Health Information’ subscale of the Health Literacy Questionnaire. Scores ranged from 0 to 20. A higher score is indicative of better medical information‐seeking ability.

The participants had a mean age of 23.4 (standard deviation [SD] = 8.8) years at the time of the interview, and slightly more than half of the participants were male (n = 110, 55.0%) (Table 2). The mean age at primary cancer diagnosis was 7.3 (SD = 5.2) years, and the average elapsed time since treatment completion was 13.4 (SD = 7.6) years. The majority of the survivors were not diagnosed with any chronic health condition at the time of interview (n = 117, 58.5%), while one‐fifth (n = 46, 23.0%) reported at least one chronic health condition. The most common chronic health conditions were neurocognitive impairment (n = 31, 15.5%), endocrine problems (n = 22, 11.0%) and hearing problems (n = 20, 10.0%) (Appendix S4).

Regarding the SES factors, approximately half of the survivors reported a monthly household income of less than HKD 30 000 (n = 90, 45%) and/or lived in government‐subsidized public housing (n = 103, 51.5%). Among the adult survivors, approximately one‐third (n = 48, 30.9%) had a secondary or lower education level. Approximately half of the adult survivors were engaged in full‐time employment (n = 86, 55%).

A slight majority of survivors reported that they kept cancer‐related medical records (n = 106, 53%). The mean (SD) medical information‐seeking ability score was 13.8 (2.7) points (range: 5 to 20 points).

### 3.2 Awareness of primary cancer diagnosis

The most common type of cancer diagnosis was haematological malignancy (n = 106, 53.0%), followed by non‐CNS tumour (n = 80, 40%) and CNS tumour (n = 14, 7%) (Table 2). A minority of the survivors (15.5%) had experienced a cancer relapse.

**FIGURE 1:**
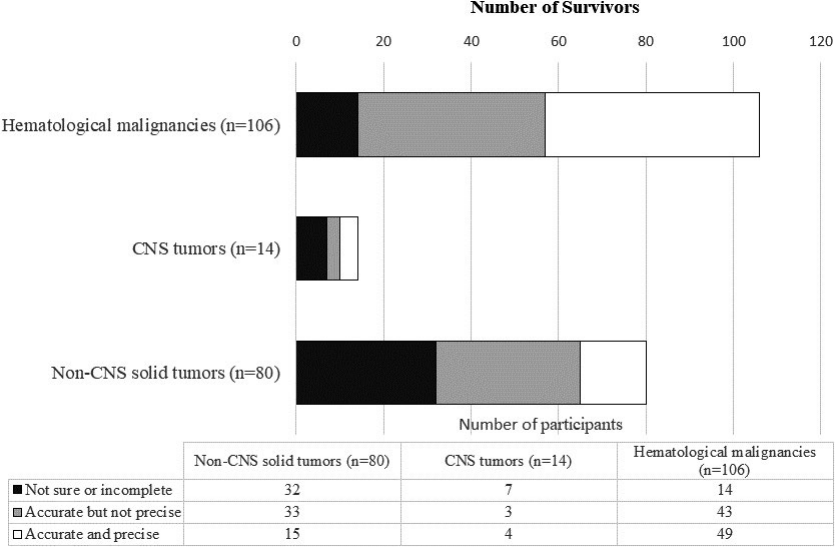
Awareness of diagnosis (n = 200). CNS, Central nervous system

Around half of the participants who were diagnosed with haematological malignancies could accurately and precisely report their diagnoses (n = 49, 46.2%) (Figure 1), whereas smaller proportions of participants with CNS tumour (n = 4, 28.5%) and non‐CNS tumour malignancies (n = 15, 18.8%) were able to do so. The majority of participants in the latter groups were unable to name the tumour sites and subtypes accurately.

### 3.3 Awareness of treatment exposure

The vast majority of survivors were treated with chemotherapy (n = 185, 92.5%) (Table 2). The most commonly used chemotherapy drugs were anthracyclines (n = 136, 68.0%), plant alkaloids (n = 125, 62.5%) and alkylating agents (n = 122, 61.0%). A minority of survivors were treated with radiotherapy (n = 70, 35.0%). Less than half underwent surgery (n = 85, 42.5%), and a minority (n = 30, 15.0%) received HSCT.

The participants reported a high level of awareness about their previous treatment modalities (chemotherapy: n = 171, 92.4%, radiation: n = 58, 82.9% and surgery: n = 75, 88.2%) (Appendix S5).

### 3.4 Awareness of potential late effects

**FIGURE 2:**
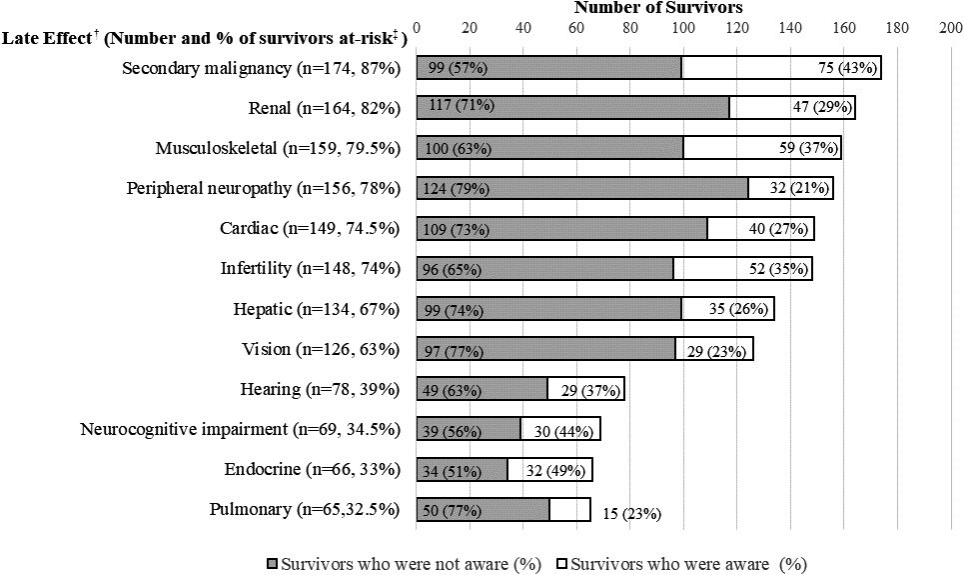
Awareness of treatment‐related late effects (n = 200). ^†^Exposure‐related health risks were determined according to the Children's Oncology Group Long‐term Follow‐up Guidelines.^17^ ^‡^Refers to the percentage of survivors at risk out of all participants (n = 200)

Based on the treatment exposures, the study cohort was at risk for a median of 6 (interquartile range: 4 to 7) of the 12 late effects evaluated in this study (Appendix S6). All survivors were at risk for at least one late effect, while 12.5% of survivors were at risk for nine or more late effects (Appendix S6). The most common at‐risk late effects were secondary malignancy (n = 174, 87.0%), renal problem (n = 164, 82.0%) and musculoskeletal problem (n = 159, 79.5%) (Figure 2).

The participants exhibited the highest levels of awareness regarding endocrine problems (n = 32, 49%), neurocognitive impairment (n = 30, 44%) and secondary malignancy (n = 75, 43%) and the lowest levels regarding peripheral neuropathy (n = 32, 21%), vision problems (n = 29, 23%) and pulmonary problems (n = 15, 23%) (Figure 2).

**FIGURE 3:**
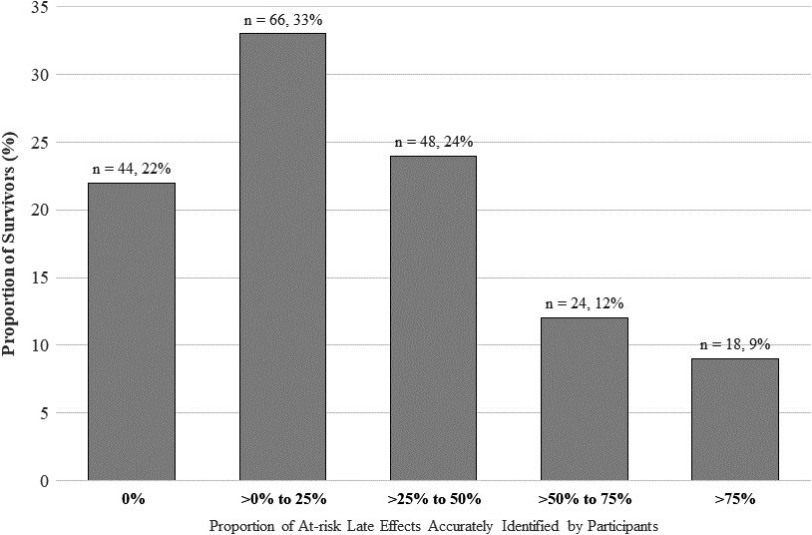
Proportion of at‐risk late effects accurately identified by participants (n = 200)

Only 9% (n = 18) of the participants could identify more than three‐quarters of the late effects for which they were at risk (Figure 3). More than half (n = 110, 55%) could only recognize less than a quarter of the potential late effect risks, and nearly a quarter (n = 44, 22%) could not identify any of their health risks (Figure 3).

### 3.5 Factors associated with cancer‐related health literacy

The univariate analysis identified the age at diagnosis, time elapsed since treatment, cancer diagnosis type, relapse status, presence of chronic health condition, medical record‐keeping habit and highest educational attainment (for adult survivors only) (Appendix S7) as factors associated with awareness.

The multivariable analysis (Table 3) showed that after adjusting for sex and age at evaluation, an older age at diagnosis was significantly associated with a better awareness of the treatment history (*B* = 0.63, 95% CI = 0.30 to 1.22). Compared with survivors of haematological malignancies, those of CNS tumours (*B* = −9.33, 95% CI= −13.41 to −5.26) and non‐CNS solid tumours (*B* = −8.47, 95% CI = −12.39 to −4.94) had a significantly lower level of knowledge about their diagnosis. Survivors who had developed a chronic health condition had significantly better awareness of diagnosis, treatment and late effects (all *P* < .05).

**TABLE 3:** Factors Associated with Cancer‐related Health Literacy (n = 200)

	Awareness of Diagnosis^a^		Awareness of Treatment^a^		Awareness of Late Effect^a^	
Score: Mean (SD) range: 0 to 100 points	67.6 (28.7)		91.3 (22.0)		28.9 (20.5)	
Demographic and clinical factors	**B (95% CI)**	***P*** *	**B (95% CI)**	***P*** *	**B (95% CI)**	***P*** *
Age at Diagnosis (years)^b^	0.42 (0.36‐1.20)	.28	0.63 (0.30‐1.22)	.**037**	0.61 (−0.16‐1.39)	.11
Time off Treatment (years)^b^	−0.16 (−0.70‐0.37)	.55	−0.24 (−0.65‐0.16)	.24	−0.16 (−0.70‐0.37)	.54
Diagnosis						
Haematological malignancies	Ref		Ref		Ref	
CNS tumours	−9.33 (−13.41 – −5.26)	**<.0001**	−0.57 (−3.85‐2.71)	.73	−1.70 (−0.59‐2.54)	.43
Non‐CNS solid tumours	−8.47 (−12.39 – −4.94)	**<.0001**	−0.37 (−4.57‐3.75)	.98	−2.68 (−1.59‐3.28)	.24
Relapse status						
No	Ref	.41	Ref	.41	Ref	.065
Yes	4.68 (−6.57‐15.94)		3.52 (−5.00 −12.04)		10.43 (−0.66‐21.53)	
Chronic health condition						
No	Ref	.**009**	Ref	.**041**	Ref	.**005**
Yes	10.87 (2.78‐18.96)		6.51 (0.26‐12.76)		11.57 (3.55‐19.60)	
Socioeconomic factors						
Highest education level^c^						
Secondary school or below	Ref	.19	Ref	.78	Ref	.069
Post‐secondary school or above	3.24 (−1.71‐8.18)		0.55 (−3.46‐4.56)		4.37 (−0.35‐9.11)	
Behavioural factors						
Medical record‐keeping habit						
None	Ref	**<.0001**	Ref	.**046**	Ref	.51
Any	16.91 (9.14‐24.68)		6.21 (0.12‐12.28)		2.65 (−5.39‐10.70)	
Medical information‐seeking habit^b^, ^d^	0.90 (−0.58‐2.39)	.23	−0.38 (−1.53‐0.75)	.50	1.48 (0.01‐2.95)	.**048**

Abbreviations: 95% CI: confidence interval; B: Unstandardized estimate; CNS: central nervous system; Ref: reference group.

^a^ A higher score is indicative of better awareness. Models are adjusted for sex and age at evaluation.

^b^ Refer to factors that were analysed as a continuous variable.

^c^ Analysis was conducted in adult survivors only.

^d^ Refers to the ‘appraisal of health information’ subscale of the Health Literacy Questionnaire. A higher score is indicative of better medical information‐seeking habit.

* Boldface: Refers to statistical significance of *P* < .05.

Survivors who had developed a medical record‐keeping habit had significantly higher scores regarding the awareness of diagnosis (*B* = 16.91, 95% CI = 9.14 to 24.68) and treatment (*B* = 6.21, 95% CI = 0.12 to 12.28), compared with those who had not developed this habit. A better medical information‐seeking habit (ie higher HLQ score) was associated with a better awareness of late effects (*B* = 1.48, 95% CI = 0.01 to 2.95). We did not identify any significant associations between SES factors and cancer‐related health literacy in the multivariable analysis.

Similar predictive factors (age at diagnosis, CNS tumour diagnosis, chronic health conditions, medical record‐keeping habit and medical information‐seeking habit) were identified in the sensitivity analysis consisting adult survivors only (Appendix S8).

## 4 DISCUSSION

This study aimed to evaluate the level and factors associated with cancer‐related health literacy among a sample of relatively young survivors of childhood cancer in Hong Kong. We found that survivors demonstrated deficient knowledge regarding treatment‐related late effects. Survivors who were diagnosed at a younger age and/or with CNS or non‐CNS solid tumour malignancies reported poorer cancer‐related knowledge. Participants who retained their personal medical records exhibited better knowledge of their condition and treatment exposures. Better medical information‐seeking habits were associated with an increased awareness of late effects. Taken together, our findings could help to identify subgroups of patients who might require more intensive education and empowerment during the survivorship phase. Developing targeted interventions for this special population may pave the way for improved rehabilitation care for survivors, which is recognized internationally as an essential component of the cancer care continuum.

Generally, participants in this study could report their cancer diagnoses and identify their major therapeutic exposures fairly accurately. This might be because the majority of the survivors treated at the study site received a ‘medical alert card’ that includes their cancer diagnoses and major treatment modalities. The oncologists reviewed the information on the card periodically during their LTFU consultation. However, they exhibited a substantially lower awareness of the potential late effects. On average, the survivors in our study could only recognize 30% of the late effects for which they were at risk. This finding is not surprising. Studies in the United States have reported similar low baseline rates of awareness of late effects (20% to 40%) among survivors of cancer.^13^, ^15^, ^16^ The paediatric oncologists in Hong Kong typically provide brief information on late effects to patients and caregivers during the discussion of cancer treatment plan. However, the low awareness of late effects in our participants suggested that such information should be reinforced throughout the cancer care continuum. Certain chronic health conditions, such as cardiopulmonary complications and secondary malignancies, may not be symptomatic or clinically apparent for years after treatment completion.^3^ Therefore, it is crucial to provide on‐going risk‐based care for survivors of childhood cancer. It is well established that survivors of childhood cancer are often lost to follow‐up from their primary paediatric clinics due to their growing independence and mobility as they advance into adulthood.^23^ Putting this into context, the LTFU clinic at the study site had an average default rate of 10% to 15%. A vast majority of survivors in our study cohort lacked knowledge about their potential risk of cardiac, pulmonary and hepatic problems. This is concerning, as survivors are likely to seek medical intervention only when these late‐occurring conditions become symptomatic, at which point life‐long treatments may be required. Our findings reiterate the urgent need to educate survivors about their personal health risks and encourage them to engage proactively in screening practices and develop health‐protective behaviours to reduce the risk of late effects.

The participants generally expressed a low level of awareness about all 12 specific late effects explored in this study. They were most familiar with the risks of endocrine problems and neurocognitive impairment, which are the two most prevalent chronic conditions in this study cohort. These conditions have a relatively earlier onset and are likely symptomatic with clinical presentation, especially in patients with CNS tumours and/or those who received cranial radiation. This probably explains why survivors who had developed a chronic health condition had better knowledge of their treatment exposure and potential late effects. Notably, we observed a significantly lower level of awareness about infertility among our cohort of local survivors (35.1%), as compared with the cohorts in the United States and Canada described by Landier et al (63%) and Syed et al (73%), respectively^13^ A similar trend was also observed in a Japanese study, in which up to half of the adolescent and young adult survivors were unaware of the risks of infertility and the options for fertility preservation before they received cancer treatment.^24^ In general, low recognition of infertility in society was a major barrier to promoting fertility preservation among childhood and adolescent cancer survivors in Asian societies.^25^ In Hong Kong, traditional Chinese childbearing attitudes are deeply rooted in the Chinese culture,^26^ although trends are changing with the current younger generation of parents. Furthermore, patients with cancer may experience psychological distress and negative emotions resulting from threatened infertility, which may persist from diagnosis through to survivorship.^27^ Accordingly, our results suggest that fertility information should be provided to parents of children with cancer as a standard practice, both during active treatment and when survivors reach reproductive age. While oncologists should communicate with patients regarding infertility risk early during the cancer care continuum, it may not be possible for them to be well‐equipped with knowledge on fertility preservation treatments. Instead, oncologists should have ready access to referrals to fertility specialists or endocrinologists.

Poorer awareness of previously received treatments might be attributable to the limited cognitive abilities of survivors during active therapy, especially those who were diagnosed at a younger age. Survivors of solid malignancies may also find it difficult to understand specific details about the tumour subtype, site and stage, which would explain their substantially less precise and accurate reports of their cancer diagnoses, compared with those of survivors of haematological malignancies. Moreover, terms like ‘Ewing's sarcoma’, ‘Wilm's tumor’ and the differential diagnosis of CNS tumours are difficult to express in Cantonese (the native dialect of Hong Kong), especially for survivors with low literacy level. They are often direct phonetic translation of the English terms and are not intuitive to a layperson (eg *wai yi mou si zung lau* for Wilm's tumour). For these survivors, parents may often be the primary sources of information regarding cancer treatment, and a failure to transmit such important information can lead to challenges. Consequently, the survivors of childhood cancer may enter adulthood without adequate knowledge about their complicated medical history. To address this, age‐appropriate counselling could be targeted at young survivors as they transition from adolescence into young adulthood. However, in most Chinese cities (eg Hong Kong), survivors with stable health typically attend the LTFU clinic only yearly or at longer intervals.^14^, ^28^ Therefore, some institutions in the United States and Europe are now adopting a ‘shared‐care’ model, in which care for survivors is coordinated between oncology specialists and primary care provider (PCP) generalists.^29^, ^30^ Survivors with more complex health conditions and the highest cancer recurrence risk level (eg recipients of HSCT) would receive closer monitoring by oncology specialists, whereas those with a limited risk of late effects would be seen by PCP generalists in the community. PCP generalists would therefore play a primary role in the basic risk‐based surveillance of late effects and on‐going health promotion, thereby helping survivors to achieve independence and a shared responsibility for their health as they transition into adulthood.

We found that survivors who reported better medical information‐seeking habits tended to have a higher level of awareness of late effects, although this association was weak and only marginally statistically significant. This finding suggests that empowering a survivor's ability to seek information about their health conditions, health risks and health promotional measures may enhance their uptake of cancer‐related knowledge. The on‐going digital revolution has made such initiatives especially relevant, as high‐functioning patients are relying increasingly on electronic health information and online platforms.^31^ For example, the government of Hong Kong is currently promoting the use of the ‘eHealth’ mobile application,^32^ which allows registered users to view parts of their health records (eg medications, vaccine records). In this context, individuals with a low educational attainment, lower SES and poorer internet skills are at a disadvantage and might require help to appraise and identify reliable sources of information. Guidelines are now in place to help the general community gauge the quality of online health information by using rating scales such as the Quality Index for health‐related Media Reports (QIMR),^33^ the ‘Date, Author, References, Type, and Sponsor’ (DARTS) tool^34^ and the QUality Evaluation Scoring Tool (QUEST).^35^ Specific to paediatric oncology, we suggest that local survivors would benefit from the existing resources developed by international groups. Since May 2020, the COG has launched traditional and simplified Chinese versions of the Healthlinks patient education materials,^17^ a set of publicly available authoritative resources on late effects that were written in a native Chinese language. Such initiative may help to bridge the identified knowledge gap and empower Chinese survivors of childhood cancer as they take up age‐appropriate ownership of their health.

Encouragingly, we found that a medical record‐keeping habit was associated with better health literacy among the study participants. This finding provides an important basis for the development of a personalized ‘Survivorship Care Plan’ (SCP) during the early phase of a patient's cancer survivorship journey.^17^, ^36^ Emerging studies are now supporting the benefits of SCPs in terms of improving survivors’ comprehension of health information and facilitating communication between survivors, oncologists and PCPs.^37^, ^38^, ^39^ For example, one study demonstrated that the provision of an SCP and professional counselling can effectively motivate survivors of childhood cancer who were treated with cardiotoxic therapies to undergo cardiomyopathy screening.^40^ Currently, we are incorporating individualized counselling on personal health risks into a centralized survivorship programme for local survivors of childhood cancer in Hong Kong. Our future work will include evaluating the short‐term effects of this intervention on the survivors’ awareness of their personal health risks, and the long‐term effects such as sustained efficacy and changes in health behaviours (eg engaging in cancer screening practices and physical activity).

### 4.1 Limitations

This study is subject to the following limitations. First, only survivors who attended the clinic's LTFU consultations were interviewed. It is reasonable to assume that our study participants might be more health conscious than survivors who failed to visit the LTFU clinic. The distribution of cancer diagnoses of survivors in the LTFU is generally reflective of the survival profile of childhood cancers in Hong Kong. This might explain why there was a higher proportion of leukaemia survivors (39%) and lower proportion of CNS tumour survivors (7%) in this study sample, as compared to the expected proportions of leukaemia and CNS tumour cases in Hong Kong based on the public cancer registry (around 27% and 12%, respectively).^1^ Notably, the education attainment of adult survivors (69% with post‐secondary education) seemed to be slightly higher than the proportion estimate in the age‐matched general population (57% of individuals aged 25 to 34 years old with post‐secondary education).^41^ This limitation may have introduced sampling bias, reduced the validity of our findings and led to an overestimation of the survivors’ health literacy. Although the study tool was designed based on published studies, the psychometric properties might not sufficiently measure the variables of interest. Furthermore, we did not collect other variables that may influence health literacy, which is a complex and multifactorial construct. For example, robust evidence from the literature demonstrates that socioeconomic factors are powerful determinants of health‐related outcomes and main causes of health disparities. However, we did not identify any significant relationship between the SES and cancer‐related health literacy, though a post hoc analysis revealed that higher household income and education attainment were associated with better (non‐cancer‐specific) medical information‐seeking abilities (Appendix S9). The few indicators of SES adopted in our study might not have been sensitive enough to detect differences in the awareness outcomes. We did not conduct a comprehensive measure of SES in the context of Hong Kong; such an investigation should also include information about the housing tenure, occupation, monthly expenditures and economic hardship.^42^ The actual rates of chronic health conditions may be underestimated due to the high rates of undiagnosed disease and inconsistent screening of health conditions in survivors. However, we tried to capture chronic health conditions comprehensively by including both clinical records and self‐reported data. Admittedly, the latter approach might be subjected to inaccurate reporting but it is still recognized to be effective in facilitating collection of health outcomes data in epidemiological studies involving paediatric cancer population.^43^, ^44^ Despite these limitations, the current study provides evidence to support the development of targeted interventions and facilitate larger‐scale research agendas intended to improve the health and psychosocial outcomes of survivors of childhood cancer in Hong Kong.

## 5 CONCLUSION

The results of our study reveal a deficiency in cancer‐related knowledge among long‐term survivors of childhood cancer in Hong Kong, especially those diagnosed with solid tumours and/or at a younger age. The awareness of treatment‐related late effects was generally poor in this population. Our findings highlight the need to facilitate communication with survivors about the potential treatment‐related late effects and develop tailored educational interventions and behavioural interventions to improve their health statuses. As the majority of survivors are still adolescents and young adults in the early period of survivorship, helping these individuals to advocate for themselves could potentially improve their health and psychosocial outcomes and will eventually translate to a substantial positive impact on society.

## CONFLICTS OF INTEREST

All authors have disclosed no conflicts of interest.

## AUTHOR CONTRIBUTIONS

Lok Sum Yang and Chung Tin Ma contributed to study design, acquisition of data, data analysis, drafting of manuscript, administrative support and critical revision for important intellectual content. Chun Him Chan, Mei Shum Luk and Hoi Kei Woo contributed to study design, acquisition of data, data analysis, drafting of manuscript and critical revision for important intellectual content. Vivian Wing‐yan Lee, Alex Wing Kwan Leung, Samantha Lai‐ka Lee and Nelson Chun‐yiu Yeung contributed to study design, data interpretation and critical revision for important intellectual content. Chi Kong Li contributed to study design, data interpretation, clinical supervision and critical revision for important intellectual content. Yin Ting Cheung contributed to solicit funding, study design, acquisition of data, data analysis, drafting of manuscript, administrative support and critical revision for important intellectual content.

## Supporting information

Appendix S1‐S9

## Data Availability

The data that support the findings of this study are available from the corresponding author upon reasonable request.
