# Supplementary material for: Awareness of diagnosis, treatment and risk of late effects in Chinese survivors of childhood cancer in Hong Kong
Source: Health Expect. 2021 Jun 8;24(4):1473–86. doi: 10.1111/hex.13288 (PMC8369092; doi:10.1111/hex.13288)
Supplement: Supplementary file 1 — Appendix S1‐S9 [file HEX-24-1473-s001.docx]

**Appendices**

Appendix S1: Outline of Questionnaire and Responses

| Objectives | Questions (response options) |
| --- | --- |
| Section 1:  Demographic and socioeconomic information | 1. Date of birth 2. Sex 3. Interview date 4. Level of education (Primary, Secondary/Higher Diplomacy/Bachelor/Master or above/Other tertiary) 5. [Caregiver only] Relationship with survivor 6. Employment status (Student/Full-time employment/Part-time employment/Housewife/Unemployed/Retired) 7. Health insurance status (Yes/No) 8. Monthly household income (<$15,000/$15,001-$30,000/$30.001-$50,000/>$50,000) 9. Housing (Public housing/Home Ownership Scheme housing/Private housing/Others: village housing, mansion, hostel) 10. Family cancer history (Yes/No)  - Familial proximity (First-degree relative/Second-degree relative/Distant relative)  1. Medical record keeping (Printed discharge summaries/Self-recorded notes/Others/None) |
| Section 2:  Awareness of cancer diagnosis^†^ | 1. Diagnosis  - Date of diagnosis - Date of treatment completion  1. Relapse status (yes/no)  - Date of relapse - Date of treatment completion  1. [Caregiver only] The survivor’s awareness of the diagnosis (Yes/No)  - If yes, what has been informed? - If no, why not? |
| Section 3:  Awareness of treatment exposure^†^ | 1. Treatment modalities used (Chemotherapy/RT/HSCT/Surgery/Others/Not sure) 2. Radiation site (Brain/Head and face/Neck/Chest/Spine/Abdomen/Pelvis/Upper extremities/Lower extremities/Total body irradiation) 3. Surgery (Neurosurgery involving the brain/Other organs or sites) 4. Hematopoietic stem cell transplant |
| Section 4:  Awareness of late effects^†^ | 1. At-risk Late effect (Aware/Not aware)  - Pulmonary - Cardiac - Hepatic - Renal - Musculoskeletal - Vision - Hearing - Secondary malignancy - Neurocognitive impairment - Infertility - Peripheral neuropathy - Endocrine problems |
| Section 5:  Medical information-seeking habit^‡^ | (Strongly disagree, disagree, agree, strongly agree)  Items are not presented due to copyright issues. |

^†^ Adapted from Landier et al, Kadan-Lottick et al and Syed et al.^13, 15, 16^

^‡^ Refers to the “appraisal of health information” subscale of the Health Literacy Questionnaire.

Appendix S2: Scoring Rubric for Cancer-related Health Literacy

| Subscales | Grading | | | Score |
| --- | --- | --- | --- | --- |
| Awareness of cancer diagnosis | Site of cancer  (1 point)  e.g. Blood cancer  e.g. Bone tumor | Cancer type  (1 point)  e.g. Leukemia  e.g. Ewing’s sarcoma | Additional information: Site of tumor, genetic subtype, cancer subtype etc.  (1 point, if any)  e.g. Acute lymphoblastic B-cell leukemia  e.g. Ewing’s sarcoma (left upper chest wall) | Total 3 points, then convert to the scale of 100  Range: 0 to 100 points |
| Awareness of treatment exposure | Number of correctly identified treatment modalities divided by the actual number of treatment modalities  (chemotherapy, radiation therapy, surgery and hematopoietic stem cell transplant) | | | Convert to the scale of 100  Range: 0 to 100 points |
| Awareness of late effects | Number of correctly identified late effect risks divided by the actual number of late effects at-risk of  Exposure-related health risks were determined according to the Children’s Oncology Group Long-term Follow-up Guidelines. | | | Convert to the scale of 100  Range: 0 to 100 points |

Appendix S3: Recruitment Flowchart


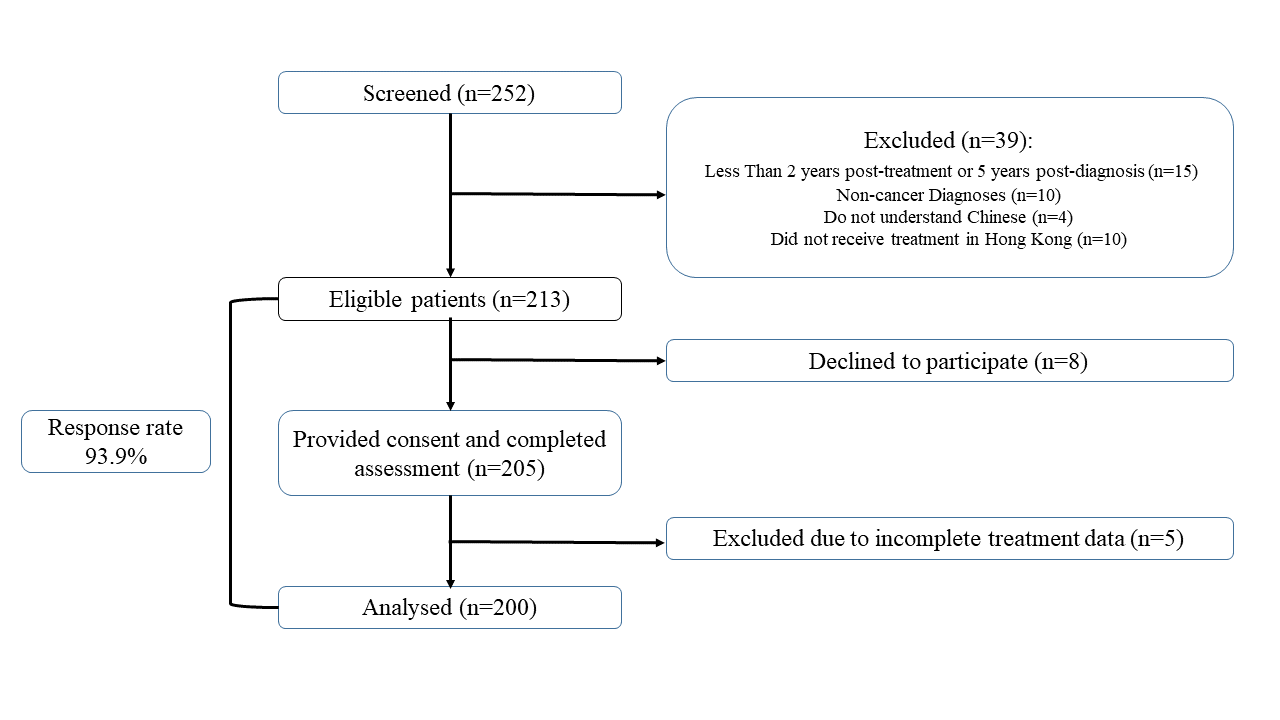


Appendix S4: Types of Chronic Health Conditions (n = 200)

| Late Effect ^†^ | n | % |
| --- | --- | --- |
| Secondary malignancy | 2 | 1.0 |
| Renal problems | 6 | 3.0 |
| Musculoskeletal problems | 14 | 7.0 |
| Peripheral neuropathy | 15 | 7.5 |
| Cardiac problems | 10 | 5.0 |
| Infertility | 8 | 4.0 |
| Hepatic problems | 3 | 1.5 |
| Vision problems | 14 | 7.0 |
| Hearing problems | 20 | 10.0 |
| Neurocognitive impairment | 31 | 15.5 |
| Endocrine problems | 22 | 11.0 |
| Pulmonary problems | 9 | 4.5 |
| Any^‡^ | **83** | **41.5** |

^†^ Information on chronic health conditions was collected through self- or proxy- reports, as well as verification with the Statistical Classification of Diseases and Related Health Problems- [ICD]-9 codes) and doctors’ consultation notes registered on the Clinical Management System (CMS). Only health conditions with a reported age of onset after the completion of treatment were included in the analysis.

^‡^ Numbers do not add up to 200 as some survivors may have more than 1 chronic health condition

Appendix S5: Awareness of Treatment Modalities (n = 200)

| Treatment Modality | Number of survivors who had received each treatment modality (%) * | Number of participants who reported accurately (%) ^‡^ |
| --- | --- | --- |
| Chemotherapy  Radiation  Surgery  Hematopoietic stem cell transplant | 185 (92.5)  70 (35.0)  85 (42.5)  30 (15.0) | 171 (92.4)  58 (82.9)  75 (88.2)  29 (96.7) |

* Refers to the proportion of survivors who had been treated with each treatment modality (Denominator refers to the overall cohort i.e. n = 200)

^‡^ Refers to the proportion of participants who accurately reported the treatment modality that the survivor had received

Appendix S6: Number of At-Risk Late Effects (n = 200)

| Number of At-risk Late Effects | Number of Survivors | Proportion (%) |
| --- | --- | --- |
| 1 | 9 | 4.5 |
| 2 | 14 | 7.0 |
| 3 | 22 | 11.0 |
| 4 | 24 | 12.0 |
| 5 | 27 | 13.5 |
| 6 | 28 | 14.0 |
| 7 | 30 | 15.0 |
| 8 | 21 | 10.5 |
| 9 | 10 | 5.0 |
| 10 | 7 | 3.5 |
| 11 | 5 | 2.5 |
| 12 | 3 | 1.5 |
| Median (interquartile range) | 6 (4 – 7) |  |

Appendix S7: Univariate Analysis of Factors Associated with Cancer-related Health Literacy (n = 200)

|  | Awareness of Diagnosis^†^ | | Awareness of Treatment^†^ | | Awareness of Late Effect^†^ | |
| --- | --- | --- | --- | --- | --- | --- |
| Mean (SD) range 0 to 100 points | 67.6 (28.7) |  | 91.3 (22.0) |  | 28.9 (20.5) |  |
| Demographic and clinical factors |  |  |  |  |  |  |
|  | ***r*** | ***P**** | ***r*** | ***P**** | ***r*** | ***P**** |
| Age at Diagnosis (years)^‡^ | 0.06 | 0.393 | 0.13 | **0.062** | 0.15 | **0.033** |
| Time off Treatment (years)^‡^ | 0.03 | 0.668 | -0.14 | **0.037** | 0.03 | 0.594 |
|  | **Mean scores (95% CI)** | ***P**** | **Mean scores**  **(95% CI)** | ***P**** | **Mean scores (95% CI)** | ***P**** |
| Diagnosis  Hematological malignancies  CNS tumors  Other solid tumors | 76.1 (70.9-81.3)  57.1 (38.0-76.3)  58.3 (52.4-64.3) | **<0.0001** | 91.4 (86.9-95.8)  95.2 (88.2-102.2)  90.6 (85.7-95.5) | 0.21 | 30.3 (24.7-35.8)  31.7 (17.1-46.3)  26.6 (20.2-33.1) | 0.36 |
| Relapse status  Yes  No | 72.0 (60.6-83.5)  66.9 (62.6-71.2) | 0.29 | 94.9 (90.3-99.5)  90.7 (87.1-94.2) | 0.76 | 38.6 (27.1-50.0)  27.1 (22.9-31.4) | **0.033** |
| Chronic health condition  No  Yes | 63.2 (58.2-68.3)  73.8 (67.6-79.5) | **0.009** | 88.7 (83.8-93.0)  94.9 (90.9-97.9) | **0.048** | 24.0 (19.0-29.6)  35.7 (30.3-41.0) | **0.004** |
| Socioeconomic factors |  |  |  |  |  |  |
|  | **Mean scores (95% CI)** | ***P**** | **Mean scores**  **(95% CI)** | ***P**** | **Mean scores (95% CI)** | ***P**** |
| Highest education level^§^  Secondary school or below  Post-secondary school or above | 59.0 (50.0-68.0)  68.5 (62.5-73.8) | **0.066** | 86.6 (77.9-93.9)  91.3 (86.9-95.0) | 0.25 | 24.3 (17.1-31.3)  32.1 (26.3-37.9) | **0.087** |
| Employment status^§^  Full-time  Non-full time | 66.1 (58.4-73.5)  65.1 (59.2-71.4) | 0.82 | 92.4 (87.7-96.3)  86.7 (80.3-92.7) | 0.14 | 31.7 (25.0-38.9)  28.1 (22.6-30.3) | 0.43 |
| Household income  Above HKD 30,000 (USD 3,850)  HKD 30,000 or lower | 68.8 (64.1-73.4)  65.6 (57.3-74.0) | 0.35 | 91.0 (87.4-94.7)  93.9 (89.0-98.8) | 0.80 | 29.6 (25.2-34.1)  27.1 (16.0-38.2) | 0.46 |
| Housing type  Private  Public | 69.4 (63.6-75.3)  65.7 (60.1-71.3) | 0.29 | 92.0 (87.5-96.5)  90.6 (86.3-95.0) | 0.48 | 29.9 (24.2-35.6)  28.3 (22.6-34.0) | 0.47 |
| Private health insurance  No  Yes | 67.0 (61.5-72.4)  68.6 (62.6-74.6) | 0.65 | 89.2 (84.8-93.6)  94.0 (89.7-98.3) | 0.13 | 28.7 (23.5-33.9)  28.3 (22.2-34.5) | 0.78 |
| Behavioral factors |  |  |  |  |  |  |
|  | **Mean scores (95% CI)** | ***P**** | **Mean scores**  **(95% CI)** | ***P**** | **Mean scores (95% CI)** | ***P**** |
| Medical record keeping habit  None  Any | 58.9 (53.1-64.7)  75.5 (70.3-80.7) | **<0.0001** | 88.1 (82.9-93.3)  94.2 (90.6-97.7) | **0.035** | 27.8 (21.9-33.7) | 0.42 |
|  | ***r*** | ***P*** | ***r*** | ***P*** | ***r*** | ***P*** |
| Medical information-seeking ability ^‡ ¶^ | 0.08 | 0.22 | -0.09 | 0.16 | 0.14 | **0.048** |

95% CI: Confidence interval; CNS: Central nervous system

^†^ A higher score is indicative of better awareness.

^‡^ Association is presented using Spearman correlation coefficient (*r*)

^§^ Analysis was conducted in adult survivors only

^¶^ Refers to the “appraisal of health information” subscale of the Health Literacy Questionnaire. A higher score is indicative of better medical information-seeking ability.

***** Boldface: Refers to statistical significance of *P*<0.1. Factors that are associated with any of the awareness scales at *P*<0.1 were included in subsequent multivariable analysis.

Appendix S8: Factors Associated with Cancer-related Health Literacy in Adult Survivors (n = 155) Sensitivity Analysis

|  | Awareness of Diagnosis^†^ | | Awareness of Treatment^†^ | | Awareness of Late Effect^†^ | |
| --- | --- | --- | --- | --- | --- | --- |
| Score: Mean (SD) range: 0 to 100 points | 65.6 (29.7) |  | 89.9 (23.9) |  | 29.7 (28.4) |  |
| Demographic and clinical factors | **B (95% CI)** | ***P**** | **B (95% CI)** | ***P**** | **B (95% CI)** | ***P**** |
| Age at Diagnosis (years)^‡^ | 1.03 (0.71 – 1.99) | **0.035** | 1.30 (0.54 – 2.06) | **0.001** | 0.88 (-0.042 – 1.81) | 0.061 |
| Time off Treatment (years)^‡^ | -0.59 (-1.46 – 0.27) | 0.17 | -0.67 (-1.37 – 0.02) | 0.059 | -0.78 (-1.61 – 0.05) | 0.065 |
| Diagnosis  Hematological malignancies  CNS tumors  Non-CNS solid tumors | Ref  -9.84 (-14.63 – -5.04)  -8.56 (-11.48 – -3.92) | **<0.0001**  **<0.0001** | Ref  -0.10 (-3.97 – 4.18)  -0.28 (-3.28 – 3.39) | 0.96  0.82 | Ref  -2.48 (-7.32 – 2.36)  -2.14 (-1.89 – 3.13) | 0.31  0.45 |
| Relapse status  No  Yes | Ref  6.94 (-5.77 – 19.64) | 0.28 | Ref  4.79 (-5.47 –15.07) | 0.41 | Ref  11.00 (-1.13 – 23.14) | 0.075 |
| Chronic health condition  No  Yes | Ref  10.78 (1.24 – 20.32) | **0.027** | Ref  8.64 (0.93 – 16.34) | **0.028** | Ref   1. 62 (3.52 – 21.72) | **0.007** |
| Socioeconomic factors | **B (95% CI)** | ***P**** | **B (95% CI)** | ***P**** | **B (95% CI)** | ***P**** |
| Highest education level^§^  Secondary school or below  Post-secondary school or above | Ref  3.24 (-1.71 – 8.18) | 0.19 | Ref  0.55 (-3.46 – 4.56) | 0.78 | Ref  4.37 (-0.35 – 9.11) | 0.069 |
| Behavioral factors | **B (95% CI)** | ***P**** | **B (95% CI)** | ***P**** | **B (95% CI)** | ***P**** |
| Medical record keeping habit  None  Any | Ref  19.58 (10.45 – 28.72) | **<0.0001** | Ref  7.65 (0.05 – 15.36) | **0.050** | Ref  1.13 (-8.15 – 10.42) | 0.81 |
| Medical information-seeking habit ^‡ ¶^ | 0.87 (-0.74 – 2.48) | 0.28 | -0.40 (-1.71 – 0.90) | 0.54 | 1.41 (0.01 – 3.37) | **0.050** |

B: Unstandardized estimate; 95% CI: Confidence interval; CNS: Central nervous system; Ref: Reference group

^†^ A higher score is indicative of better awareness. Models are adjusted for sex and age at evaluation

^‡^ Refer to factors that were analyzed as a continuous variable

^§^ Analysis was conducted in adult survivors only

^¶^ Refers to the “appraisal of health information” subscale of the Health Literacy Questionnaire. A higher score is indicative of better medical information-seeking habit.

***** Boldface: Refers to statistical significance of *P*<0.05.

Appendix S9: Post-Hoc Analysis on Socioeconomic Factors and Medical Information-seeking Habit

|  | **Mean score (95% CI)**^‡^ | ***P*** |
| --- | --- | --- |
| **Highest education level^§^**  Secondary school or below  Post-secondary school or above | 21.2 (20.4 – 22.0)  22.1 (21.3 – 22.9) | **0.032** |
| **Employment status^§^**  Full-time  Non-full time | 21.8 (21.0 – 22.6)  21.7 (20.9 – 22.5) | 0.718 |
| **Household income**  Above HKD 30,000 (USD 3,850)  HKD 30,000 or lower | 22.1 (21.4 – 22.8)  20.2 (19.2– 21.1) | **<0.01** |
| **Housing type**  Private  Public | 22.4 (21.7 – 23.1)  21.1 (20.2 – 22.0) | **0.024** |
| **Private health insurance**  No  Yes | 21.3 (20.6 – 22.0)  22.2 (21.4 – 23.1) | 0.070 |

95% CI: Confidence interval

^†^ The rationale behind this post-hoc analysis is identify socioeconomic factors associated with non-cancer specific, general medical-information seeking habit. The Mann–Whitney U test was used to compare mean scores between groups defined by each socioeconomic variable.

^‡^ Refers to the “appraisal of health information” subscale of the Health Literacy Questionnaire. Total score ranged from 5 to 20. A higher score is indicative of better medical information-seeking ability.

^§^ Analysis was conducted in adult survivors only
